# Supplementary material for: Pituitary–Adrenal Axis and Peripheral Immune Cell Profile in Long COVID
Source: Biomedicines. 2024 Mar 5;12(3):581. doi: 10.3390/biomedicines12030581 (PMC10968573; doi:10.3390/biomedicines12030581)
Supplement: Supplementary file 1 [file biomedicines-12-00581-s001.zip › biomedicines-2874749-supplementary.pdf]

Supplementary data

Pituitary-adrenal axis and peripheral immune cell profile in Long COVID

Jaume Alijotas-Reig (1,2,3), Ariadna Anunciacion-Llunell (1), Enrique Esteve-Valverde (4), Stephanie Morales-Pérez (5), Sergio Rivero-Santana (5), Jaume Trapé (6,7), Laura Gonzalez-Garcia (6,7), Domingo Ruiz (5), Joana Marques-Soares (1,2,3), Francesc Miro-Mur (1)

(1) Systemic Autoimmune Diseases Research Unit, Vall d'Hebron Institut de Recerca (VHIR), Barcelona 08035, Catalonia, Spain.

(2) Systemic Autoimmune Diseases Unit, Department of Internal Medicine, Hospital Universitari Vall d'Hebron (HUVH), 08035 Barcelona, Catalonia, Spain.

(3) Department of Medicine, Faculty of Medicine, Universitat Autònoma de Barcelona (UAB), 08035 Barcelona, Catalonia, Spain.

(4) Systemic Autoimmune Diseases Unit, Department of Internal Medicine, Hospital Universitari Parc Taulí, 08208 Sabadell, Catalonia, Spain.

(5) Internal Medicine Department, Althaia Healthcare University Network of Manresa, Systemic Autoimmune Disease Unit, 08243 Manresa, Catalonia, Spain.

(6) Laboratory Medicine, Althaia Healthcare University Network of Manresa 08243 Manresa, Catalonia, Spain.

(7) Tissue Repair and Regeneration Laboratory (TR2Lab), Institut de Recerca i Innovació en Ciències de la Vida i de la Salut a la Catalunya Central (IrisCC), 08500 Vic, Catalonia, Spain

Corresponding authors: jaume.alijotas@vallhebron.cat (J.A.-R.); francesc.miro@vhir.org (F.M.-M.); Tel.: +34934894194 (J.A.-R.); +34934894047 (F.M.-M.)

Supplementary tables

Table S1. Comparison of clinical parameters between Long COVID and recovered patients.

Parameter	Reference value	Long-COVID No	Long-COVID Yes	P-value
Haemoglobin (g/dL)	12.0-15.0	14.4 [1.7]	14.2 [2.1]	0.67
Leukocytes (x10e9/L)	4.0-11.0	5.7 [1.44]	5.6 [1.34]	0.96
Platelets (x10e9/L)	140.0-400.0	257 [88]	211 [50]	0.26
ESR (mm/h)	0.0-25.0	12 [11]	10 [12]	0.21
PT (ratio)	0.7-1.2	1 [0.06]	1 [0.11]	0.73
aPTT (ratio)	0.7-1.2	1 [0.01]	1 [0]	0.76
D-dimer (ng/mL)	0.0-240.0	82 [43.25]	76 [75]	0.39
TSH (mUI/L)	0.37-4.7	2.25 [1.47]	1.59 [1.29]	0.26
ACTH (µg/dL)	18.0-25.0	13 [4.75]	17.5 [11.75]	0.53
NT-proBNP (pg/mL)	0.5.0-30.0	25.5 [6.2]	35.6 [33.2]	0.15
C3 (mg/dL)	88.0-201.0	109 [15.35]	103.15 [24.08]	0.35
C4 (mg/dL)	15.0-45.0	22.8 [6.75]	23.75 [8.07]	0.71
Creatinine (mg/dL)	0.51-0.95	0.67 [0.26]	0.7 [0.24]	0.61
GF (mL/min/1.73m2)	90.0-120.0	99.27 [18.53]	96.56 [15.3]	0.88
Urate (mg/dL)	2.6-6.0	5.2 [1.55]	4.5 [1.65]	0.68
Cholesterol (mg/dL)	132.0-240.0	202 [23]	193 [39.5]	0.43
HDL (mg/dL)	40.0-100.0	51 [20]	52 [13.25]	0.62
LDL (mg/dL)	0.0-130.0	121 [41]	111 [39]	0.63
Triglyceride (mg/dL)	43.0-200.0	77 [38]	106 [63]	0.61
Protein (g/dL)	6.6-8.3	6.49 [0.27]	6.59 [0.44]	0.16
Albumin (g/dL)	3.5-5.2	3.91 [0.15]	4.1 [0.25]	0.08
ALT (IU/L)	7.0-35.0	17 [5]	16.5 [15.5]	0.74
AST (IU/L)	10.0-35.0	15 [4]	18 [6]	0.08
GGT (IU/L)	6.0-38.0	18 [13]	20.5 [21.5]	0.75
Calcium (mg/dL)	8.8-10.6	8.79 [0.44]	9.02 [0.28]	0.16
Magnesium (mg/dL)	1.9-2.5	1.97 [0.23]	2.02 [0.21]	0.95
LDH (IU/L)	120.0-246.0	163 [27.5]	164.5 [18.25]	0.80
CK (IU/L)	0.0-174.0	94 [61.5]	97 [74]	0.98
Ferritin (ng/mL)	25.0-250.0	93 [119]	123.5 [132.75]	0.61
CRP (mg/L)	0.0-10.0	2.1 [5.6]	1.55 [3.7]	0.66
RF (IU/mL)	0.0-14.0	9.7 [2.53]	9.05 [1.92]	0.31
ACE-2 (mmol/mL/min)	0.0-40.0	25.95 [15.93]	23.9 [14.5]	0.83
IgM (mg/dL)	40.0-230.0	94.5 [49.75]	108 [62]	0.43
IgG (mg/dL)	700.0-1600.0	949.5 [298]	957 [229.75]	0.70
IgA (mg/dL)	70.0-400.0	244 [149.7]	194.5 [140.75]	0.18
IgE (kU/L)	0.0-150.0	13.5 [12.2]	26.2 [54.92]	0.24

Data is median [interquartile range (IQR)]. ESR, erythrocyte sedimentation rate; PT, prothrombin time; aPTT, activated partial thromboplastin time; TSH, thyrotropin; NT-proBNP, N-terminal pro-B type natriuretic peptide; C3 complement component 3; C4, complement component 4; GF, glomerular filtrate; HDL, low density lipoproteins; HDL, high density lipoproteins; ALT, alanine amino transferase; AST, aspartate amino transferase; GGT, gamma-glutamyl transferase; LDH, lactate dehydrogenase; CK creatine kinase; CRP, C-reactive protein; RF, rheumatoid factor; ACE2, angiotensin converting enzyme-2; Ig, immunoglobulin. Mann-Whitney U test showed no differences in any clinical laboratory variable between individuals with and without Long COVID.

Table S2. Long COVID symptoms in participants with Long COVID or not.

	**Long COVID**		
**Variables**	**No (13)**	**Yes (29)**	**p-value**
Abdominal cramps	1 (7.69)	1 (3.45)	0.53
Ageusia	0 (0)	3 (10.34)	0.54
Alternate bowel movements	1 (7.69)	0 (0)	0.31
Anosmia	0 (0)	2 (7.14)	1.00
Anxiety	1 (7.69)	17 (58.62)	0.0024
Artharlgia	0 (0)	16 (55.17)	0.0005
Fatigue	2 (15.38)	28 (96.55)	0.0000
Cefalea	0 (0)	6 (20.69)	0.15
Depressive symptoms	2 (15.38)	13 (44.83)	0.09
Diarrhea	1 (7.69)	0 (0)	0.31
Dyspnea	0 (0)	14 (48.28)	0.0016
Fever of unknown origin	0 (0)	1 (3.45)	1.00
Lack of concentration	1 (7.69)	12 (41.38)	0.0358
Loss of voice	0 (0)	2 (6.9)	1.00
Memory loss	1 (7.69)	10 (34.48)	0.13
Myalgia	1 (7.69)	18 (62.07)	0.0018
Paresthesia	0 (0)	2 (6.9)	1.00
Persistent cough	0 (0)	9 (31.03)	0.0382
Persistent sore throat	0 (0)	8 (27.59)	0.0427
Rhinitis	0 (0)	3 (10.34)	0.54
Sexual disorder	0 (0)	3 (10.34)	0.54
Thoracic pain	0 (0)	4 (13.79)	0.29
Tinnitus	0 (0)	1 (3.45)	1.00

Data are n (%). Fisher's exact test.

Table S3. Long COVID symptoms in our cohort of participants having less than 2-fold cortisol induction in the ACTH stimulation test.

	Fold cortisol		
**Variables**	**< 2 (n=12)**	**≥ 2 (n=30)**	**p-value**
Abdominal cramps	0 (0)	2 (6.9)	1.00
Ageusia	1 (7.69)	2 (6.9)	1.00
Alternate bowel movements	0 (0)	1 (3.45)	1.00
Anosmia	1 (7.69)	1 (3.57)	0.54
Anxiety	6 (46.15)	12 (41.38)	1.00
Artharlgia	6 (46.15)	10 (34.48)	0.51
Fatigue	11 (84.62)	19 (65.52)	0.28
Cefalea	2 (15.38)	4 (13.79)	1.00
Depressive symptoms	7 (53.85)	8 (27.59)	0.16
Diarrhea	0 (0)	1 (3.45)	1.00
Dyspnea	6 (46.15)	8 (27.59)	0.30
Fever of unknown origin	1 (7.69)	0 (0)	0.31
Lack of concentration	5 (38.46)	8 (27.59)	0.49
Loss of voice	1 (7.69)	1 (3.45)	0.53
Memory loss	4 (30.77)	7 (24.14)	0.71
Myalgia	8 (61.54)	11 (37.93)	0.19
Paresthesia	0 (0)	2 (6.9)	1.00
Persistent cough	8 (61.54)	1 (3.45)	0.0001
Persistent sore throat	6 (46.15)	2 (6.9)	0.0063
Rhinitis	3 (23.08)	0 (0)	0.0249
Sexual disorder	1 (7.69)	2 (6.9)	1.00
Thoracic pain	2 (15.38)	2 (6.9)	0.58
Tinnitus	1 (7.69)	0 (0)	0.31

Data are n (%). Fisher's exact test.

Table S4. Number of leukocyte subpopulations in patients with and without Long COVID.

	Long COVID		
**Cell population**	**yes**	**no**	**p-value**
B cells	135.81 [42.03]	137.24 [51.67]	0.80
T cells	894.7 [343.11]	911.97 [340.2]	0.49
CD4 T cells	519.56 [247.53]	535.87 [354.8]	0.80
CD8 T cells	257.65 [115.89]	295.6 [176.12]	0.41
CD4cm	282.64 [107.22]	245.55 [89.34]	0.49
CD4em	56.62 [49.57]	59.44 [18.49]	0.98
CD4emra	1.83 [4.17]	5.99 [6.48]	0.21
CD8cm	83.3 [42.58]	66.37 [50]	0.35

CD8em	26.27 [24.79]	37.03 [32.38]	0.27
CD8emra	24.98 [57.57]	58.24 [67.63]	0.39
ϒδ TCR cells	24.6 [16.51]	20.05 [19.7]	0.86
NK cells	126.21 [112.96]	156.54 [95.4]	0.22
NKT cells	32.35 [45.28]	32.02 [27.8]	0.83
Monocytes	354.78 [152.36]	435.21 [141.89]	0.37
Monocytes CD14hi	286.91 [134.51]	334.4 [157.33]	0.41
Monocytes CD14lo	41.41 [21.28]	51.82 [20.5]	0.22
Neutrophils	2616.98 [1289.19]	3780.34 [747.87]	0.0277
Exhausted B cells	5.66 [4.88]	3 [6.38]	0.25
Mature B cells	36.37 [19.35]	27.92 [17.15]	0.18
HLA-DR+CD38+ CD4	6.94 [4.52]	5.77 [6.25]	0.54
CD38+ CD4	67.57 [71.09]	89.58 [101.66]	0.83
HLA-DR+CD38+ CD8	9.45 [8.6]	7.61 [3.92]	0.65
CD38+ CD8	33.57 [28.16]	36.84 [26.83]	0.39
CD39+CD73+ CD4	0.17 [0.45]	0.17 [0.27]	0.30
CD39+ CD4	12.31 [12.62]	12.5 [26.88]	0.59
CD39+CD73+ CD8	0.62 [0.64]	0.49 [1.35]	0.92
CD39+ CD8	3.75 [2.73]	3.8 [5.78]	0.68
CD73+ CD4	8.34 [12.31]	5.24 [4.2]	0.19
CD73+ CD8	13.92 [17.1]	8.35 [18.03]	0.62
CD103+ CD4	4.28 [1.93]	3.66 [2.21]	0.54
CD69+ CD4	1.37 [3.66]	1 [0.89]	0.16
CD69+CD103+ CD4	0 [0.07]	0 [0]	0.27
CD103+ CD8	8.45 [5.89]	6.31 [3.54]	0.08
CD69+ CD8	1.92 [1.53]	1.31 [1.4]	0.46
CD69+CD103+ CD8	0 [0.1]	0 [0.09]	0.76
Treg cells	7.46 [7.4]	6.26 [8.23]	0.51
CD39+ CD73+ Treg cells	0.06 [0.1]	0 [0]	0.08
CD39+ Treg cells	1.46 [3.51]	1.03 [2.16]	1.00
CD73+ Treg cells	0.12 [0.26]	0.08 [0.18]	0.40
HLA-DR+CD38+ NK cells	4.57 [6.66]	2.87 [2.07]	0.59
CD38+ NK cells	61.78 [56.36]	106.86 [74.19]	0.19
CD39+CD73+ NK cells	0.53 [0.8]	0.61 [0.59]	0.70
CD69+ NK cells	0.71 [0.61]	0.95 [1.12]	0.50
HLA-DR+CD38+ ϒδ TCR cells	0.13 [0.24]	0.15 [0.6]	1.00
CD38+ ϒδ TCR cells	0.34 [0.48]	0.97 [1.32]	0.27
CD69+ ϒδ TCR cells	0.12 [0.16]	0.11 [0.1]	0.92
CD73+ B cells	42.89 [49.25]	50.21 [54.07]	0.44
CD162+ B cells	13.3 [11.18]	10.2 [6.8]	0.77
CD38+ B cells	66.98 [48.27]	80.47 [53.71]	0.22
HLA-DR+CD38+ Monocytes	247.88 [131.42]	333.77 [158.1]	0.22
CD39+ Monocytes	157.99 [73.26]	102.75 [119.93]	0.18

CD73+CD39+ Monocytes	6.58 [9.95]	6.28 [8.22]	0.54
HLA-DR+CD38+ Neutrophils	6.53 [12.19]	6.39 [18.03]	0.92

Cell number is median [interquartile range (IQR)] 10^6/L. cm, central memory; em, effector memory; emra, effector memory CD45RA positive; NK, natural killer; Treg, regulatory T cells. Mann-Whitney U-statistical test.

Table S5. Frequency of leukocyte subpopulations in patients with and without Long COVID.

	**Long-COVID**		
**Cell population**	**Yes**	**No**	**p-value**
B cells	2.97 (1.49)	2.66 (2.24)	0.36
T cells	20.85 (6.38)	17.40 (3.50)	0.0423
CD4 T cells	62.1 (14.2)	60.65 (9.50)	0.74
CD8 T cells	28.85 (13.18)	30.95 (10.3)	0.57
CD4cm	55.8 (15.55)	51.9 (10.95)	0.29
CD4em	12.6 (9.32)	12.35 (4.93)	0.71
CD4emra	0.365 (1.01)	0.94 (0.47)	0.25
CD8cm	40.00 (20.1)	27.25 (9.85)	0.09
CD8em	10.15 (9.65)	11.68 (7.58)	0.90
CD8emra	11.65 (21.42)	23.1 (30.24)	0.49
ϒδ TCR cells	2.54 (2.55)	2.21 (0.90)	0.76
NK cells	8.65 (7.27)	10.03 (9.07)	0.25
NKT cells	2.70 (3.54)	1.95 (2.96)	0.70
Monocytes	8.63 (2.48)	7.00 (2.28)	0.11
Monocytes CD14hi	79.50 (3.78)	79.40 (6.65)	0.82
Monocytes CD14lo	12.65 (4.63)	15.20 (5.24)	0.22
Neutrophils	99.05 (1.33)	98.95 (0.78)	0.63
Exhausted B cells	3.86 (4.96)	2.35 (2.17)	0.09
Mature B cells	25.80 (13.40)	17.90 (9.20)	0.0298
HLA-DR+CD38+ CD4	1.50 (0.78)	1.16 (0.31)	0.12
CD38+ CD4	13.25 (7.62)	15.30 (10.29)	0.68
HLA-DR+CD38+ CD8	2.77 (1.89)	2.24 (1.39)	0.29
CD38+ CD8	10.81 (5.08)	13.66 (7.83)	0.46
CD39+CD73+ CD4	0.04 (0.08)	0.02 (0.04)	0.14
CD39+ CD4	1.97 (2.78)	2.54 (2.76)	0.80
CD39+CD73+ CD8	0.19 (0.32)	0.21 (0.27)	0.87
CD39+ CD8	1.33 (1.12)	1.11 (0.90)	0.53
CD73+ CD4	1.72 (2.22)	0.81 (1.18)	0.21
CD73+ CD8	5.07 (5.61)	2.58 (3.56)	0.31
CD103+ CD4	0.72 (0.45)	0.67 (0.18)	0.66
CD69+ CD4	0.29 (0.36)	0.14 (0.18)	0.17
CD69+CD103+ CD4	0.00 (0.01)	0.00 (0.00)	0.20

CD103+ CD8	2.78 (1.43)	1.99 (0.48)	0.0330
CD69+ CD8	0.52 (0.60)	0.40 (0.26)	0.54
CD69+CD103+ CD8	0.00 (0.03)	0.00 (0.02)	0.73
Treg cells	0.84 (0.94)	0.72 (0.94)	0.63
CD39+ CD73+ Treg cells	0.49 (1.11)	0.00 (0.00)	0.0270
CD39+ Treg cells	37.05 (35.48)	44.89 (27.78)	0.31
CD73+ Treg cells	2.20 (2.86)	0.53 (1.98)	0.10
HLA-DR+CD38+ NK cells	3.72 (6.41)	2.36 (2.75)	0.35
CD38+ NK cells	60.45 (30.08)	72.16 (21.07)	0.59
CD39+CD73+ NK cells	0.36 (1.45)	0.39 (0.41)	0.63
CD69+ NK cells	0.54 (0.70)	0.44 (0.58)	0.94
HLA-DR+CD38+ ϒδ TCR cells	0.92 (1.66)	1.06 (2.93)	0.98
CD38+ ϒδ TCR cells	2.47 (2.93)	4.93 (4.27)	0.31
CD69+ ϒδ TCR cells	0.80 (1.26)	0.56 (0.35)	0.53
CD73+ B cells	31.50 (24.85)	24.55 (18.00)	0.89
CD162+ B cells	9.92 (6.61)	6.44 (3.38)	0.13
CD38+ B cells	53.80 (12.45)	60.45 (8.07)	0.23
HLA-DR+CD38+ Monocytes	70.10 (21.10)	73.35 (6.30)	0.78
CD39+ Monocytes	49.37 (28.20)	29.85 (20.13)	0.08
CD73+CD39+ Monocytes	1.82 (2.08)	1.52 (1.94)	0.33
HLA-DR+CD38+ Neutrophils	0.24 (0.47)	0.17 (0.45)	0.79

Data is N (%). Frequency is from its parental gating (see supplementary figure S4 for strategy of gating). cm, central memory; em, effector memory; emra, effector memory CD45RA positive; NK, natural killer; Treg, regulatory T cells. Fisher's exact test.

Table S6. Serum cytokine levels in patients with Long COVID compared with those of recovered patients.

	**Long COVID**		
**Cytokine**	**No (N=9)**	**Yes (n=25)**	**p-value**
IL-6	2.92 [2.64]	2.60 [1.40]	0.75
TNFα	0.04 [0.03]	0.06 [0.14]	0.70
IFNγ	0.24 [0.23]	0.22 [0.48]	0.82
IL-1β	0.22 [0.10]	0.34 [0.40]	0.67
IL-2	0.25 [0.29]	0.29 [0.62]	0.75
IL-10	13.97 [3.74]	20.12 [15.5]	0.11
IL-12p40	9.27 [6.75]	16.08 [29.18]	0.70
IL-12p70	0.36 [0.17]	0.54 [0.18]	0.67
GDF-8	0.50 [0.17]	0.50 [0.51]	0.75

Cytokine values are expressed as median [interquartile range (IQR)]. Units of cytokines are pg/ml, except for GDF-8 that are ng/ml. Mann-Whitney U test.

Supplementary Figures

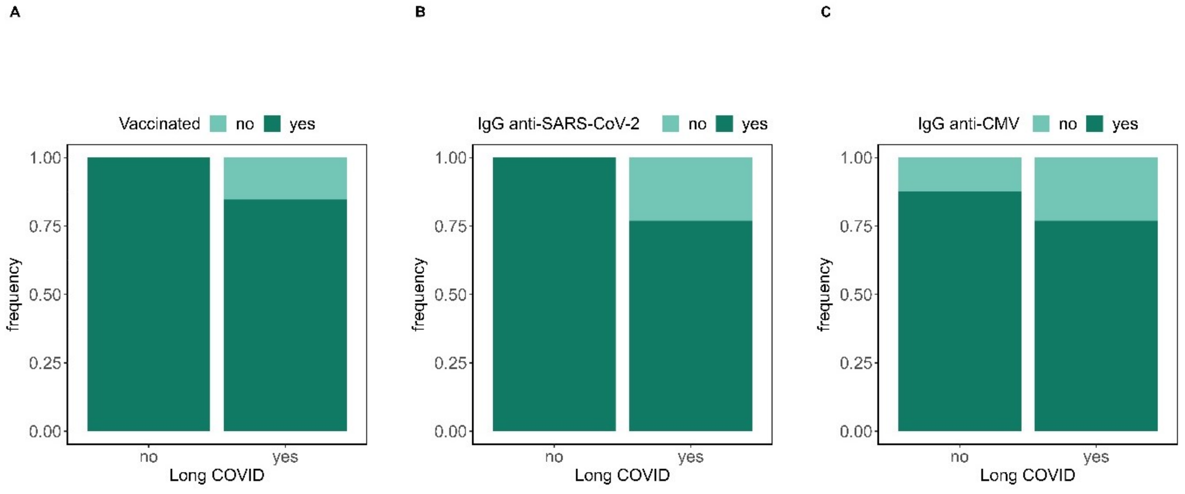

Figure S1. anti-SARS-CoV-2 vaccination rates. Frequency of vaccinated anti-SARS-CoV-2 participants (A), percentage of participants with antibodies against SARS-CoV-2 spike protein (B), or anti-CMV (C), segregated by having or not Long COVID.

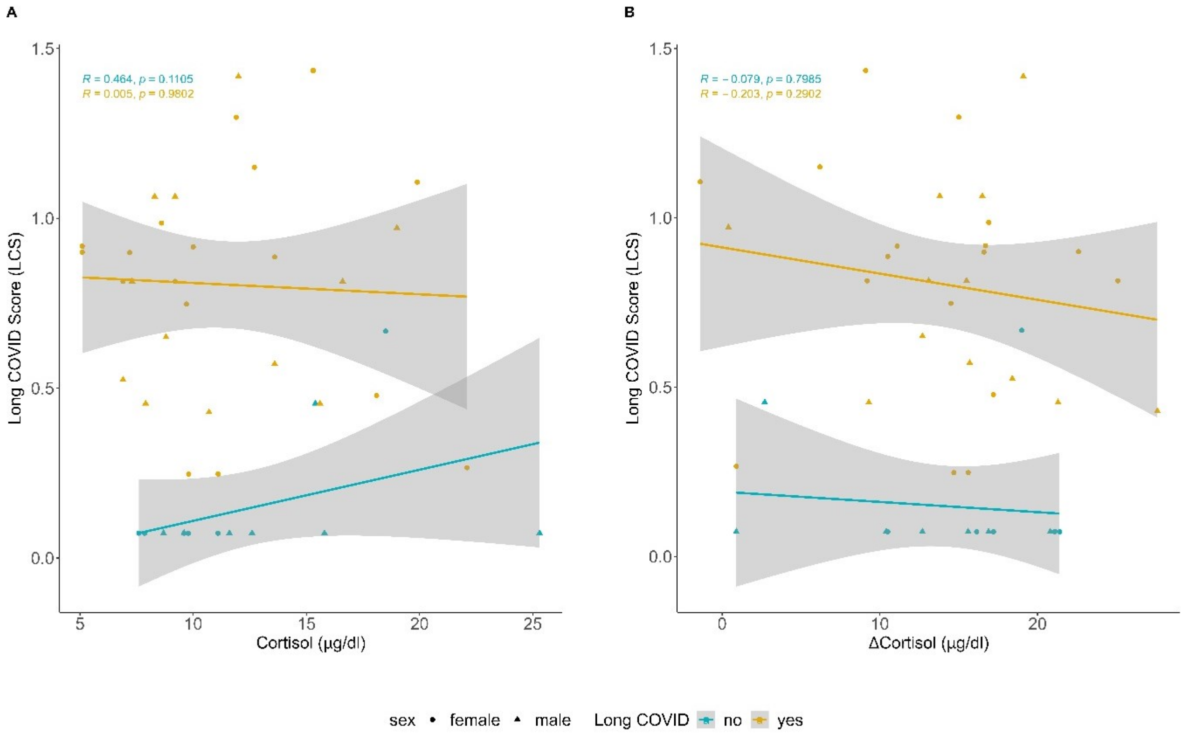

Figure S2. Association of Long COVID Score (LCS) with cortisol levels. Correlation of Long COVID Score (LCS) with basal cortisol levels (A) or ΔCortisol levels (B). Sex of participants is indicated by two different shape symbols. Spearman correlation test.

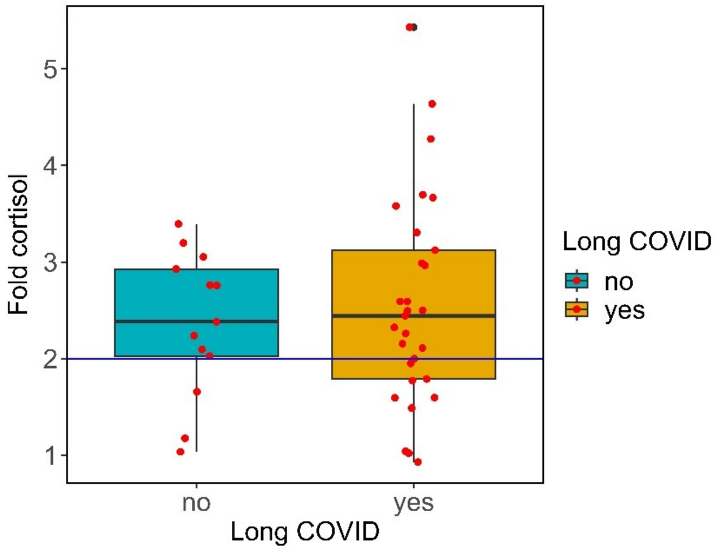

Figure S3. Fold cortisol induction in ACTH stimulation test. Fold cortisol induction after 1 hour of ACTH stimulation test between patients with Long COVID and recovered individuals. Those participants with less than a 2-fold induction are below the horizontal blue line and they are referred as non-responders to ACTH stimulation test.

Supplementary Figure S4

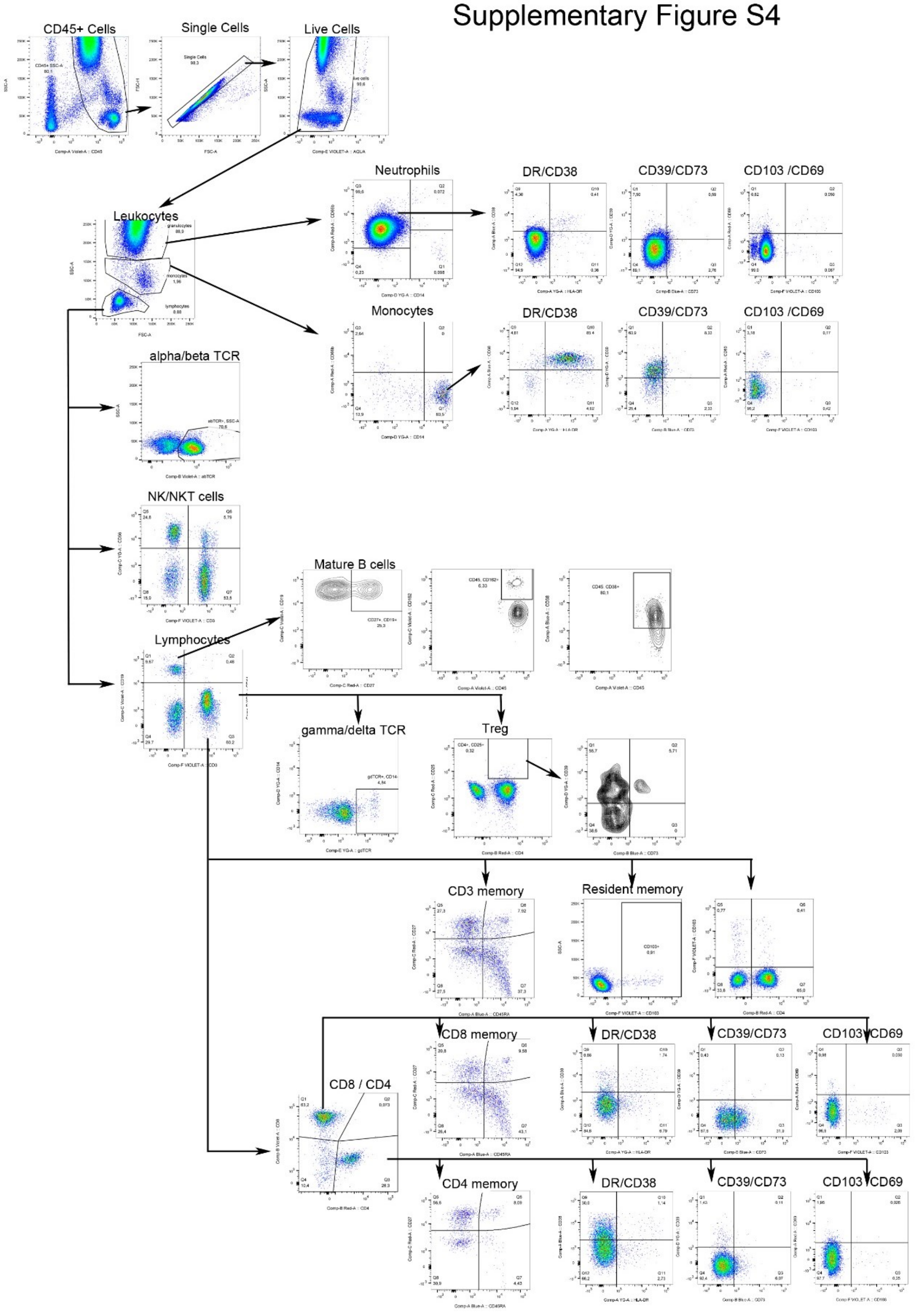

Figure S4. Strategy of gating in flow cytometry. Strategy of gating used to describe the different blood leukocyte subpopulations of our Long COVID participants.
